# Medical Faculty of the University of New York

**Published:** 1850-08

**Authors:** 


The Medical Faculty of the University of New York have published an official invitation for applications to fill the two vacant chairs in that institution, viz., the Professorship of the Principles, Practice and Operations of Surgery, and that of the Institutes and Practice of Medicine.

